# Structure–Activity Relationship of Oleanane-Type Pentacyclic Triterpenoids on Nuclear Factor κB Activation and Intracellular Trafficking and *N*-Linked Glycosylation of Intercellular Adhesion Molecule-1

**DOI:** 10.3390/ijms25116026

**Published:** 2024-05-30

**Authors:** Kaori Nakano, Yuka Yokota, Quy Van Vu, Francesca Lagravinese, Takao Kataoka

**Affiliations:** 1 Department of Applied Biology, Kyoto Institute of Technology, Matsugasaki, Sakyo-ku, Kyoto 606-8585, Japan; 2 Biomedical Research Center, Kyoto Institute of Technology, Matsugasaki, Sakyo-ku, Kyoto 606-8585, Japan

**Keywords:** nuclear factor κB, intercellular adhesion molecule-1, *N*-glycosylation, celastrol, pristimerin, oleanolic acid, moronic acid, glycyrrhetinic acid, maslinic acid, *α*-boswellic acid

## Abstract

In our previous study, two oleanane-type pentacyclic triterpenoids (oleanolic acid and maslinic acid) were reported to affect the *N*-glycosylation and intracellular trafficking of intercellular adhesion molecule-1 (ICAM-1). The present study was aimed at investigating the structure–activity relationship of 13 oleanane-type natural triterpenoids with respect to the nuclear factor κB (NF-κB) signaling pathway and the expression, intracellular trafficking, and *N*-glycosylation of the ICAM-1 protein in human lung adenocarcinoma A549 cells. Hederagenin, echinocystic acid, erythrodiol, and maslinic acid, which all possess two hydroxyl groups, decreased the viability of A549 cells. Celastrol and pristimerin, both of which possess an *α*,*β*-unsaturated carbonyl group, decreased cell viability but more strongly inhibited the interleukin-1α-induced NF-κB signaling pathway. Oleanolic acid, moronic acid, and glycyrrhetinic acid interfered with *N*-glycosylation without affecting the cell surface expression of the ICAM-1 protein. In contrast, *α*-boswellic acid and maslinic acid interfered with the *N*-glycosylation of the ICAM-1 protein, which resulted in the accumulation of high-mannose-type *N*-glycans. Among the oleanane-type triterpenoids tested, *α*-boswellic acid and maslinic acid uniquely interfered with the intracellular trafficking and *N*-glycosylation of glycoproteins.

## 1. Introduction

Pro-inflammatory cytokines, such as interleukin-1 (IL-1), are produced by macrophages and other types of cells at the onset of inflammation [1]. Pro-inflammatory cytokines stimulate vascular endothelial cells to up-regulate diverse cell surface adhesion molecules [2,3]. As one of these adhesion molecules, intercellular adhesion molecule-1 (ICAM-1) (also known as CD54) interacts with integrin family proteins, which play an essential role in the recruitment of circulating leukocytes and their extravasation [2,3]. ICAM-1 expression is also induced in other cells, such as epithelial cells, and plays an additional role in wound healing and tumor metastasis [4,5]. Human and mouse ICAM-1 proteins possess eight and ten *N*-glycosylation sites, respectively, and are highly glycosylated as other inducible adhesion molecules [6,7]. High-mannose-type *N*-glycans are generally synthesized and transferred to translating polypeptides at the endoplasmic reticulum (ER) and are then converted to mature complex-type *N*-glycans by the removal and addition of sugar chains during their transport to the cell surface [8,9]. The *N*-glycosylation of ICAM-1 is required for the cell surface expression of the ICAM-1 protein, which was previously shown to be prevented by tunicamycin [10,11]. In comparisons with major complex-type *N*-glycans of the ICAM-1 protein, minor high-mannose-type *N*-glycans more strongly enhanced the interaction between monocytes and the endothelium [12,13].

In response to pro-inflammatory cytokines, the expression of ICAM-1 was shown to be mainly up-regulated by the transcriptional activation of the *ICAM1* gene [14,15]. Among multiple transcription factors, nuclear factor κB (NF-κB) family proteins play a critical role in the up-regulation of ICAM-1 mRNA [14,15]. In unstimulated cells, NF-κB heterodimers are associated with the inhibitor of NF-κB (IκB) protein, and this tertiary complex is sequestered in the cytosol by masking the nuclear localization signal of NF-κB subunits [16,17]. Upon stimulation with IL-1, the IκB protein is phosphorylated by the IκB kinase complex and subsequently degraded by the ubiquitin–proteasome system, which allows for the translocation of NF-κB heterodimers from the cytosol to the nucleus and the subsequent transcriptional activation of NF-κB target genes [18,19].

Pentacyclic triterpenoids are major constituents in many plants and exert diverse biological effects, including anti-cancer and anti-inflammatory activities [20,21,22]. Pentacyclic triterpenoids are classified into different groups, including oleanane-type, ursane-type, and lupane-type triterpenoids. In our previous study, it was reported that oleanane-type, ursane-type, and lupane-type triterpenoids isolated from *Nerium oleander*, a small tree cultivated worldwide, interfered with the expression of ICAM-1 induced by pro-inflammatory cytokines [23,24]. Based on these findings, the mechanisms by which pentacyclic triterpenoids affected the protein expression, intracellular trafficking, and *N*-glycosylation of ICAM-1 were investigated [11,25,26,27]. These studies proposed that slight structural differences in pentacyclic triterpenoids markedly affected their inhibitory mechanisms. Regarding oleanane-type triterpenoids, oleanolic acid and maslinic acid were shown to exert different effects on the *N*-glycosylation and intracellular trafficking of ICAM-1 [25,26]. However, the structure–activity relationship of oleanane-type triterpenoids remained unclear. Therefore, in this study, the biological activities of 13 oleanane-type triterpenoids, including oleanolic acid and maslinic acid, were evaluated.

## 2. Results

### 2.1. Structural Features of 13 Oleanane-Type Pentacyclic Triterpenoids

**Figure 1:**
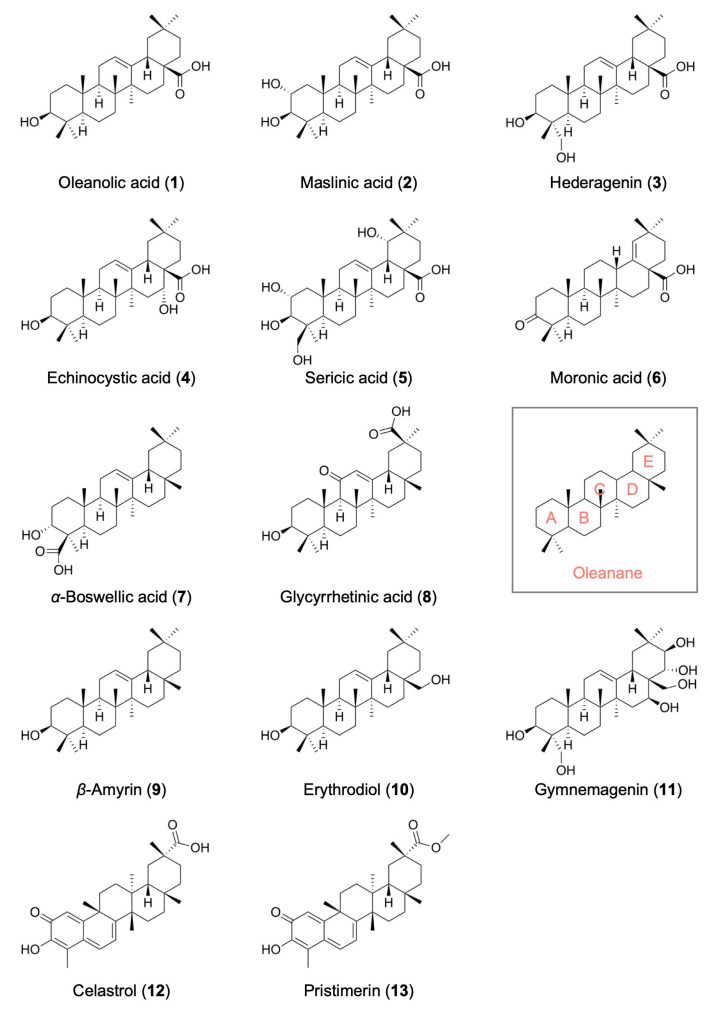
Structures of oleanane-type pentacyclic triterpenoids. Oleanane consists of rings A to E. The structures of 13 oleanane-type pentacyclic triterpenoids (numbered from **1** to **13**) are shown.

Human lung adenocarcinoma A549 cells were responsive to multiple pro-inflammatory cytokines and were stimulated to express NF-κB-responsive gene products [28]. As model cells substituted for primary vascular endothelial cells, A549 cells were selected to elucidate the mechanisms of action of diverse small-molecule compounds (including triterpenoids) on the NF-κB-dependent signaling pathway and gene expression. In the present study, 13 oleanane-type pentacyclic triterpenoids were selected for biological evaluation (Figure 1). The E ring with two methyl groups attached to the same carbon atom is the common structure of oleanane-type triterpenoids. Among these triterpenoids, oleanolic acid (**1**) is one of the triterpenoids possessing a carboxyl group and a hydroxyl group. Glycyrrhetinic acid (**8**), celastrol (**12**), and pristimerin (**13**) possess one *α*,*β*-unsaturated group.

### 2.2. Effects of Oleanane-Type Pentacyclic Triterpenoids on Cell Viability

**Figure 2:**
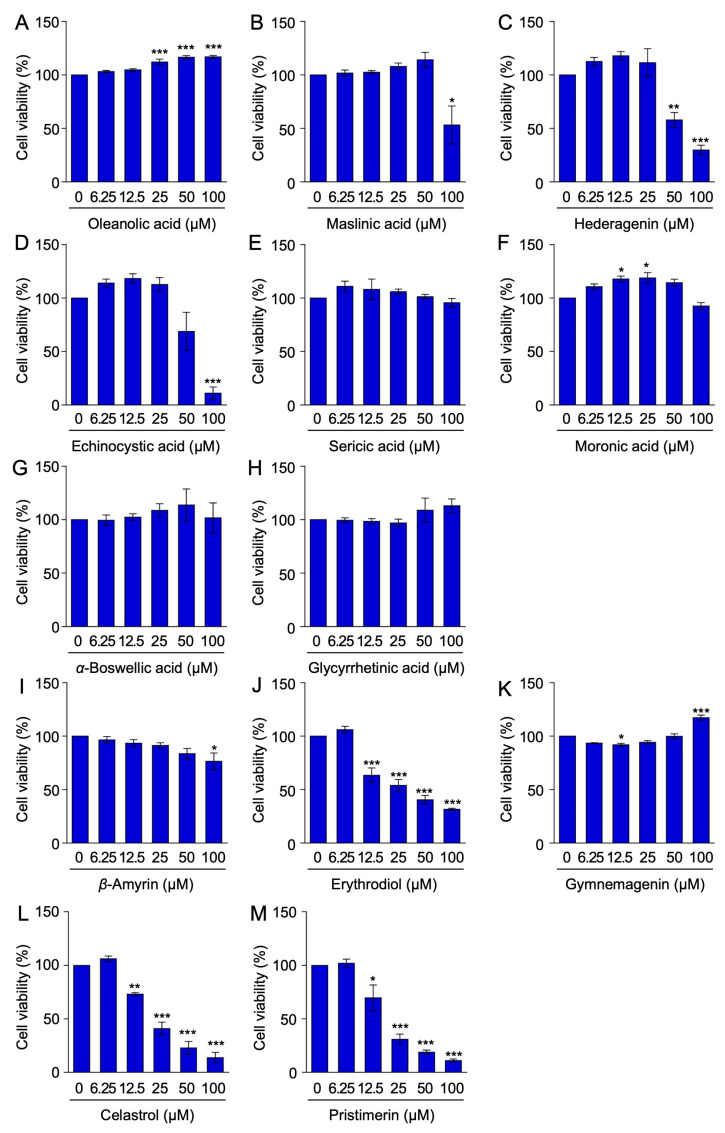
Effects of oleanane-type pentacyclic triterpenoids on cell viability. (**A**–**M**) A549 cells were incubated with serial dilutions of oleanolic acid (**1**) (**A**), maslinic acid (**2**) (**B**), hederagenin (**3**) (**C**), echinocystic acid (**4**) (**D**), sericic acid (**5**) (**E**), moronic acid (**6**) (**F**), *α*-boswellic acid (**7**) (**G**), glycyrrhetinic acid (**8**) (**H**), *β*-amyrin (**9**) (**I**), erythrodiol (**10**) (**J**), gymnemagenin (**11**) (**K**), celastrol (**12**) (**L**), and pristimerin (**13**) (**M**) for 7 h in the presence or absence of these triterpenoids. Cell viability (%) is shown as the mean ± S.E. of three (**A**,**B**,**D**–**M**) or four (**C**) independent experiments. * p < 0.05, ** p < 0.01, and *** p < 0.001, significantly different from the control.

To evaluate the biological activities of oleanane-type pentacyclic triterpenoids, their effects on cell viability were initially investigated. A 7-h incubation with a series of twofold dilutions showed that five triterpenoids, oleanolic acid (**1**), sericic acid (**5**), moronic acid (**6**), *α*-boswellic acid (**7**), and glycyrrhetinic acid (**8**), did not markedly decrease the viability of A549 cells at concentrations up to 100 µM (Figure 2A,E–H), whereas *β*-amyrin (**9**) significantly decreased cell viability at 100 µM (Figure 2I). In contrast, gymnemagenin (**11**) at 100 µM significantly increased cell viability (Figure 2K). Four triterpenoids decreased the viability of A549 cells in a dose-dependent manner (Figure 2C,J,L,M). The viability of A549 cells was reduced by erythrodiol (**10**), celastrol (**12**), and pristimerin (**13**) at concentrations higher than 12.5 µM (Figure 2J,L,M) and by hederagenin (**3**) at concentrations higher than 50 µM (Figure 2C). Maslinic acid (**2**) and echinocystic acid (**4**) decreased cell viability at 100 µM (Figure 2B,D).

### 2.3. Celastrol and Pristimerin Diminished IL-1α-Induced ICAM-1 Protein Expression

**Figure 3:**
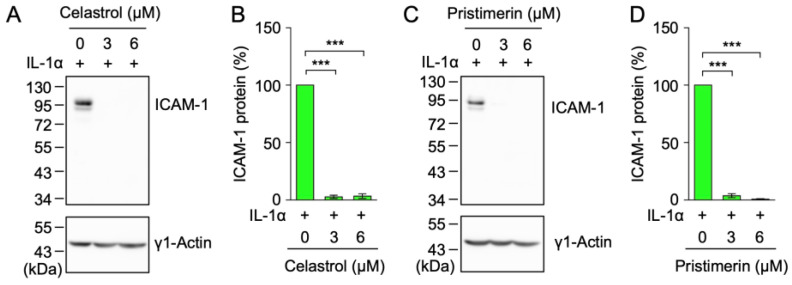
Celastrol and pristimerin reduced the IL-1α-induced ICAM-1 protein expression. (**A**–**D**) A549 cells were treated with or without celastrol (**12**) (**A**,**B**) and pristimerin (**13**) (**C**,**D**) for 1 h and were then stimulated with (+) IL-1α (0.25 ng/mL) for 6 h in the presence or absence of these triterpenoids at the indicated final concentrations. Blots are representative of three independent experiments (**A**,**C**). The amount of the ICAM-1 protein was normalized to that of the γ1-actin protein. The IL-1α stimulation (+) without compounds (0 µM) was set to 100%. The ICAM-1 protein (%) is shown as the mean ± S.E. of three independent experiments (**B**,**D**). *** p < 0.001.

Compounds possessing an *α*,*β*-unsaturated carbonyl group undergo the Michael reaction to modify the thiol groups of cysteines and thereby exert diverse biological effects [28,29]. Celastrol (**12**) and pristimerin (**13**) contain one *α*,*β*-unsaturated carbonyl group as a common structure (Figure 1). A549 cells were incubated with these triterpenoids for 1 h and were then stimulated with IL-1α for 6 h. ICAM-1 protein expression was detected by Western blotting. The IL-1α stimulation up-regulated the expression of the ICAM-1 protein, which was detected at a molecular size of between 81 and 113 kDa (Figure 3A,C). A quantitative analysis showed that celastrol (**12**) at 3 or 6 µM and pristimerin (**13**) at 3 or 6 µM markedly reduced the amount of the ICAM-1 protein (Figure 3B,D).

### 2.4. Celastrol and Pristimerin Inhibited the IL-1α-Induced NF-κB Signaling Pathway

**Figure 4:**
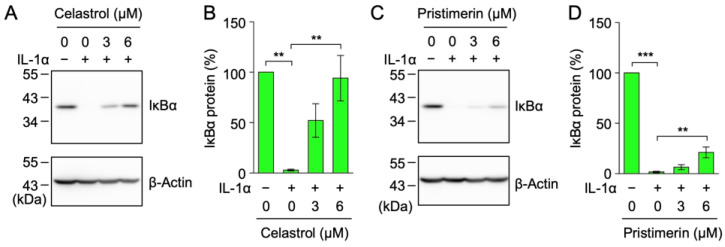
Celastrol and pristimerin inhibited the IL-1α-induced IκBα protein degradation. (**A**–**D**) A549 cells were treated with or without celastrol (**12**) (**A**,**B**) and pristimerin (**13**) (**C**,**D**) for 1 h and were then stimulated without (−) or with (+) IL-1α (0.25 ng/mL) for 15 min in the presence or absence of these triterpenoids at the indicated final concentrations. Blots are representative of three independent experiments (**A**,**C**). The amount of the IκBα protein was normalized to that of the β-actin protein. The IκBα protein (%) is shown as the mean ± S.E. of three independent experiments (**B**,**D**). ** p < 0.01 and *** p < 0.001.

ICAM-1 is one of the adhesion molecules that is mainly up-regulated by NF-κB in response to pro-inflammatory cytokines [14,15]. In the NF-κB signaling pathway, IκB kinase and/or NF-κB subunits possess cysteine residues that are essential for their biological activities, and these proteins are inhibited by many *α*,*β*-unsaturated carbonyl compounds [28,29]. Therefore, the effects of celastrol (**12**) and pristimerin (**13**) on the stimulation-dependent degradation of the IκBα protein were examined. A549 cells were treated with triterpenoids for 1 h and were then stimulated with IL-1α for 15 min. The band of the IκBα protein was clearly detectable in unstimulated A549 cells, while the IL-1α stimulation reduced the band of the IκBα protein, reflecting IκBα protein degradation (Figure 4A,C). Celastrol (**12**) and pristimerin (**13**) at a concentration of 6 µM inhibited the IL-1α-induced degradation of the IκBα protein (Figure 4A–D).

**Figure 5:**
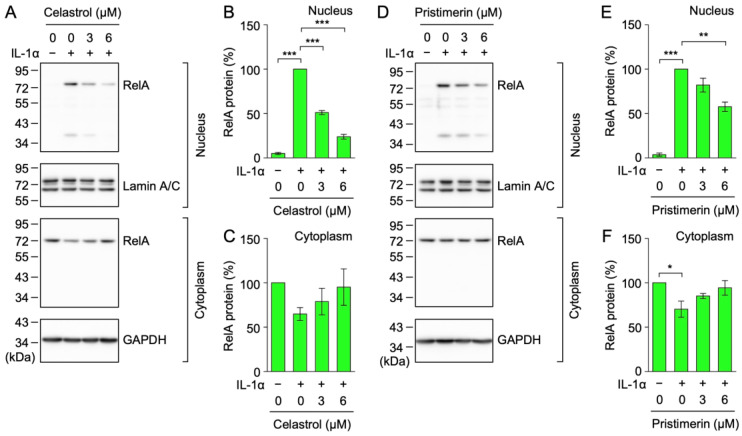
Celastrol and pristimerin inhibited the IL-1α-induced nuclear RelA translocation. (**A**–**F**) A549 cells were treated with or without celastrol (**12**) (**A**–**C**) and pristimerin (**13**) (**D**–**F**) for 1 h and were then stimulated without (−) or with (+) IL-1α (0.25 ng/mL) for 1 h in the presence or absence of these triterpenoids at the indicated final concentrations. Blots are representative of three independent experiments (**A**,**D**). The amount of the RelA protein was normalized to that of the lamin A/C protein and the glyceraldehyde-3-phosphate dehydrogenase (GAPDH) protein. The RelA protein (%) in the nucleus (**B**,**E**) and cytoplasm (**C**,**F**) is shown as the mean ± S.E. of three independent experiments. * p < 0.05, ** p < 0.01, and *** p < 0.001.

Upon IκBα degradation, NF-κB heterodimers composed of RelA (also known as p65) and p50 are translocated from the cytosol to the nucleus [18,19]. In the present study, A549 cells were treated with triterpenoids for 1 h and then stimulated with IL-1α for 1 h. The IL-1α stimulation increased the band of the RelA protein in the nucleus (Figure 5A,D). Celastrol (**12**) at 3 or 6 µM and pristimerin (**13**) at 6 µM reduced the IL-1α-induced increase in the nuclear RelA protein (Figure 5B,E). These results demonstrated that celastrol (**12**) and pristimerin (**13**) inhibited the IL-1α-induced nuclear translocation of the RelA protein. Celastrol (**12**) appeared to inhibit the NF-κB signaling pathway more strongly than pristimerin (**13**).

### 2.5. Effects of Oleanane-Type Pentacyclic Triterpenoids on IL-1α-Induced ICAM-1 Protein Expression

**Figure 6:**
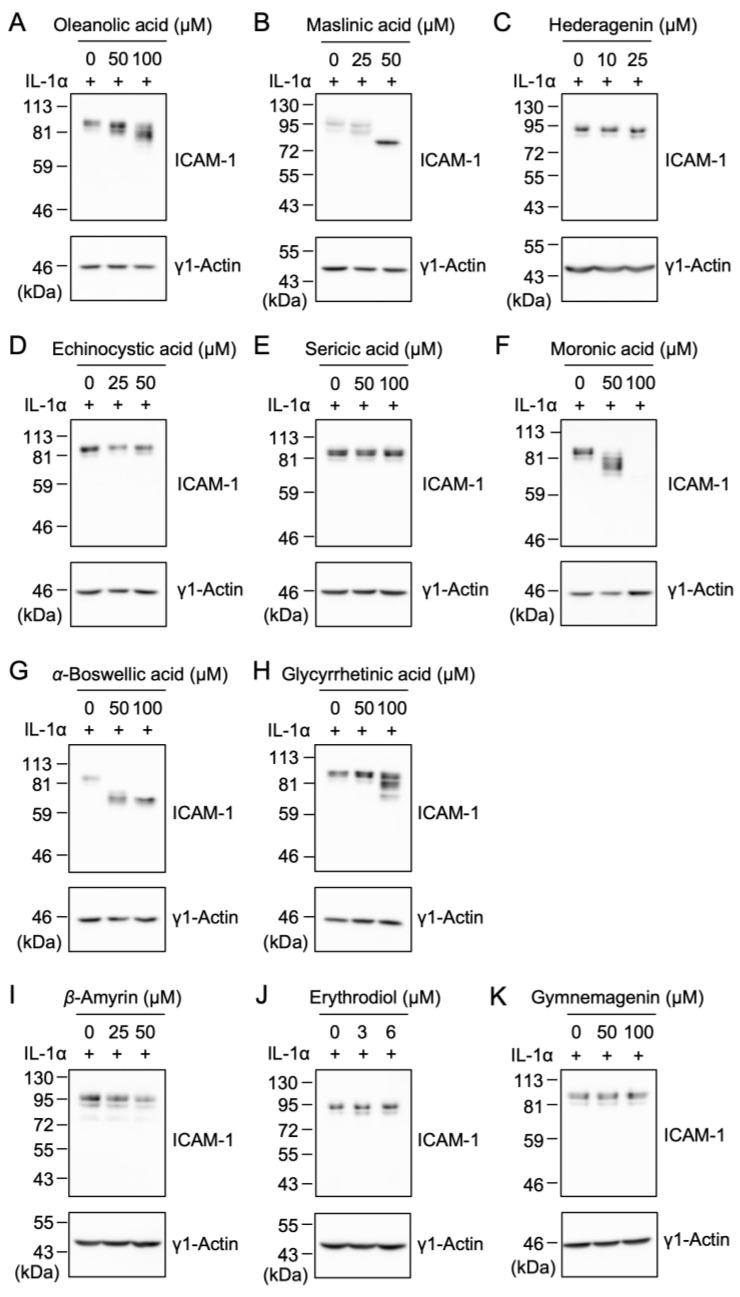
Effects of oleanane-type triterpenoids on the IL-1α-induced ICAM-1 protein expression. (**A**–**K**) A549 cells were treated with or without oleanolic acid (**1**) (**A**), maslinic acid (**2**) (**B**), hederagenin (**3**) (**C**), echinocystic acid (**4**) (**D**), sericic acid (**5**) (**E**), moronic acid (**6**) (**F**), *α*-boswellic acid (**7**) (**G**), glycyrrhetinic acid (**8**) (**H**), *β*-amyrin (**9**) (**I**), erythrodiol (**10**) (**J**), and gymnemagenin (**11**) (**K**) for 1 h and were then stimulated with (+) IL-1α (0.25 ng/mL) for 6 h in the presence or absence of these triterpenoids at the indicated final concentrations. Blots are representative of four (**A**,**D**,**F**,**G**) and three (**B**,**C**,**E**,**H**–**K**) independent experiments.

The human ICAM-1 protein is attached to eight *N*-linked glycans as a post-translational modification [4,5]. It was shown that oleanolic acid (**1**) and maslinic acid (**2**) interfered with the *N*-linked glycosylation of the ICAM-1 protein in different manners [25,26]. Therefore, the structure–activity relationship of oleanane-type pentacyclic triterpenoids on IL-1α-induced ICAM-1 protein expression were investigated by Western blotting. Consistent with our previous findings [25,26], oleanolic acid (**1**) and maslinic acid (**2**) both reduced the molecular sizes of the ICAM-1 protein (Figure 6A,B). These data newly demonstrated that moronic acid (**6**), *α*-boswellic acid (**7**), and glycyrrhetinic acid (**8**) shortened the molecular size of the ICAM-1 protein (Figure 6F–H). Hederagenin (**3**), echinocystic acid (**4**), sericic acid (**5**), *β*-amyrin (**9**), erythrodiol (**10**), and gymnemagenin (**11**) did not markedly affect ICAM-1 protein expression or its molecular sizes (Figure 6C–E,I–K). In contrast, moronic acid (**6**) at 100 µM reduced ICAM-1 protein expression (Figure 6F).

### 2.6. Effects of Moronic Acid on the IL-1α-Induced NF-κB Signaling Pathway

**Figure 7:**
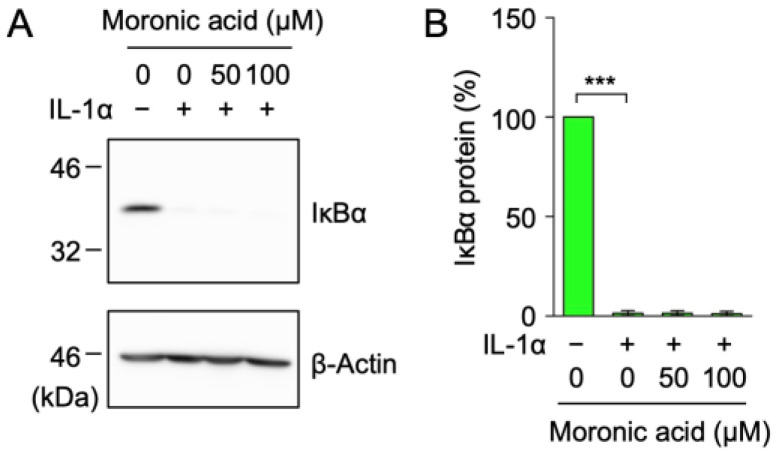
Moronic acid did not inhibit the IL-1α-induced degradation of the IκBα protein. (**A**,**B**) A549 cells were treated with or without moronic acid (**6**) (**A**,**B**) for 1 h and were then stimulated without (−) or with (+) IL-1α (0.25 ng/mL) for 15 min in the presence or absence of moronic acid (**6**) at the indicated final concentrations. Blots are representative of three independent experiments (**A**). The amount of the IκBα protein was normalized to that of the β-actin protein. The IκBα protein (%) is shown as the mean ± S.E. of three independent experiments (**B**). *** p < 0.001.

Based on the above results (Figure 6F), it was necessary to investigate whether the NF-κB signaling pathway was inhibited by moronic acid (**6**), which reduced ICAM-1 protein expression at 100 µM. A549 cells were treated with moronic acid (**6**) for 1 h and were then stimulated with IL-1α for 15 min. The IL-1α-induced degradation of the IκBα protein was not affected by moronic acid (**6**) (Figure 7A,B). These results suggest that moronic acid (**6**) did not prevent the NF-κB signaling pathway but interfered with downstream ICAM-1 transcription and translation processes.

### 2.7. Effects of Oleanane-Type Pentacyclic Triterpenoids on the IL-1α-Induced Cell Surface Expression of the ICAM-1 Protein

**Figure 8:**
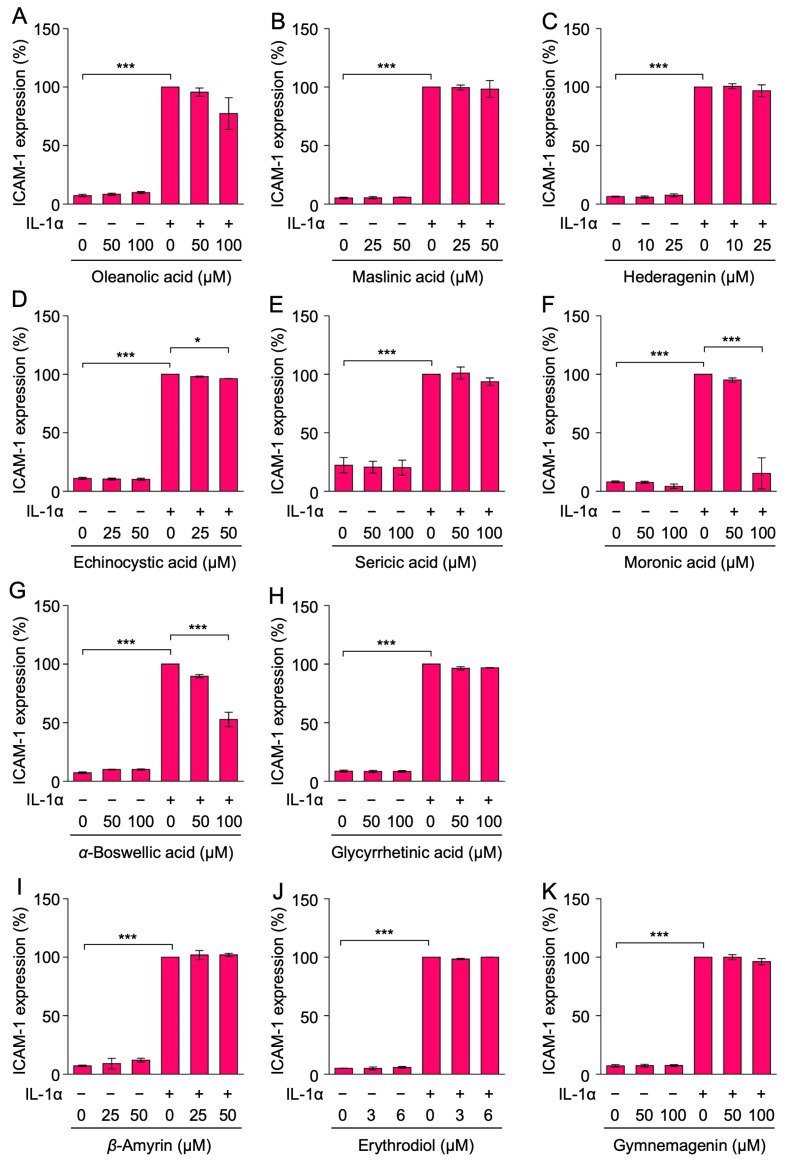
Effects of oleanane-type triterpenoids on the IL-1α-induced cell surface ICAM-1 expression. (**A**–**K**) A549 cells were treated with or without oleanolic acid (**1**) (**A**), maslinic acid (**2**) (**B**), hederagenin (**3**) (**C**), echinocystic acid (**4**) (**D**), sericic acid (**5**) (**E**), moronic acid (**6**) (**F**), *α*-boswellic acid (**7**) (**G**), glycyrrhetinic acid (**8**) (**H**), *β*-amyrin (**9**) (**I**), erythrodiol (**10**) (**J**), and gymnemagenin (**11**) (**K**) for 1 h and were then stimulated without (−) or with (+) IL-1α (0.25 ng/mL) for 6 h in the presence or absence of these triterpenoids at the indicated final concentrations. ICAM-1 expression (%) is shown as the mean ± S.E. of three (**A**–**F**,**H**–**K**) and five (**G**) independent experiments. * p < 0.05 and *** p < 0.001.

The cell surface expression of the ICAM-1 protein was assessed by cell ELISA. Consistent with previous findings [25,26], the cell surface expression of the ICAM-1 protein was not markedly affected by oleanolic acid (**1**) at 50–100 µM or maslinic acid (**2**) at 25–50 µM (Figure 8A,B). However, it was previously shown that maslinic acid (**2**) reduced the transport of the ICAM-1 protein to the cell surface at concentrations higher than 50 µM, which did not affect the viability of A549 cells [26]. *α*-boswellic acid (**7**) at 100 µM interfered with the cell surface expression of the ICAM-1 protein (Figure 8G). In contrast, hederagenin (**3**), echinocystic acid (**4**), sericic acid (**5**), glycyrrhetinic acid (**8**), *β*-amyrin (**9**), erythrodiol (**10**), and gymnemagenin (**11**) did not markedly affect the cell surface expression of the ICAM-1 protein at concentrations that did not decrease cell viability (Figure 8C–E,H–K).

### 2.8. Effects of Oleanane-Type Pentacyclic Triterpenoids on the N-Glycosylation of the ICAM-1 Protein

**Figure 9:**
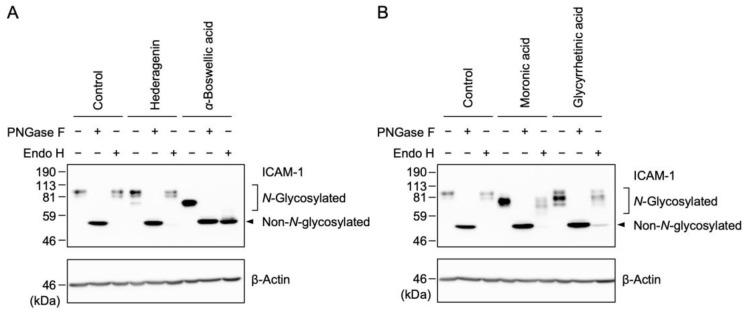
Effects of oleanane-type triterpenoids on the *N*-glycan status of the ICAM-1 protein. (**A**,**B**) A549 cells were left untreated (Control) or were treated with hederagenin (**3**) and *α*-boswellic acid (**7**) (**A**) or moronic acid (**6**) and glycyrrhetinic acid (**8**) (**B**) for 1 h and were then stimulated with IL-1α (0.25 ng/mL) for 6 h in the presence or absence of these triterpenoids. The final concentrations used were as follows: hederagenin (**3**) (50 µM), *α*-boswellic acid (**7**) (100 µM), moronic acid (**6**) (50 µM), and glycyrrhetinic acid (**8**) (100 µM). Cell lysates were treated without (−) or with (+) PNGase F or Endo H. Blots are representative of three independent experiments. Images were overexposed to show faint bands.

Endoglycosidase H (Endo H) cleaves the high-mannose-type *N*-glycans of glycoproteins, which are processed prior to the effects of Golgi α-mannosidase II. Peptide-*N*-glycosidase F (PNGase F) digests all *N*-glycans irrespective of their structures and, thus, is used to represent the position of the non-*N*-glycosylated ICAM-1 protein. It was previously shown that Endo H-resistant *N*-glycans were attached to the ICAM-1 protein in oleanolic acid (**1**)-treated cells, while Endo H-sensitive *N*-glycans were predominantly linked to the ICAM-1 protein in maslinic acid (**2**)-treated cells [25,26]. In addition to these triterpenoids, the effects of the three triterpenoids that affected the molecular sizes of the ICAM-1 protein, (i.e., moronic acid (**6**), *α*-boswellic acid (**7**), and glycyrrhetinic acid (**8**)) were examined (Figure 6F–H). In addition, the effects of hederagenin (**3**) were investigated because it shortened ICAM-1 bands at 50 µM (Figure S1). A549 cells were treated with these triterpenoids for 1 h and were then stimulated with IL-1α for 6 h. Cell lysates were prepared and subjected to treatment with Endo H or PNGase F. In *α*-boswellic acid (**7**)-treated cells, the ICAM-1 protein was converted to shorter thick bands, which were completely cleaved by Endo H to the same size as those cleaved by PNGase F in *α*-boswellic acid (**7**)-treated cells (Figure 9A), indicating that the majority of ICAM-1 proteins possessed high-mannose-type *N*-glycans only. In moronic acid (**6**)-treated cells, the thick bands of the ICAM-1 protein were mostly resistant to Endo H; however, they became slightly smaller heterogenous bands (Figure 9B), suggesting that a single ICAM-1 protein possesses a mixture of mainly Endo-H-resistant *N*-glycans and some Endo H-sensitive *N*-glycans. In glycyrrhetinic acid (**8**)-treated cells, major shortened ICAM-1 bands were resistant to Endo H, whereas shorter residual bands were cleaved by the Endo H treatment (Figure 9B). When A549 cells were treated with hederagenin (**3**), a large percentage of ICAM-1 bands were resistant to Endo H, whereas a small percentage of ICAM-1 bands with shorter molecular sizes were sensitive to Endo H (Figure 9A). Therefore, in contrast to the three other triterpenoids, it appears that hederagenin (**3**) at 50 µM weakly affects the *N*-glycosylation of the ICAM-1 protein, which may be attributed to a reduction in A549 cell viability (Figure 2C).

## 3. Discussion

In the course of our screening for anti-inflammatory agents, ursane-type, oleanane-type, and lupane-type pentacyclic triterpenoids were initially found to inhibit the cell surface expression of the ICAM-1 protein induced by pro-inflammatory cytokines [23,24]. Further experiments were conducted to investigate the molecular mechanisms by which pentacyclic triterpenoids inhibit the cell surface expression of the ICAM-1 protein induced by pro-inflammatory cytokines. Ursolic acid was shown to inhibit the intracellular trafficking of glycoproteins, including ICAM-1, from the ER to the Golgi apparatus [11]. The biological activities of three structural isomers of pentacyclic triterpenoids with the same molecular formula (C_30_H_48_O_3_) but possessing different E ring structures, oleanolic acid (**1**) (oleanane-type), ursolic acid (ursane-type), and betulinic acid (lupane-type), were studied. In contrast to ursolic acid, oleanolic acid (**1**) and betulinic acid interfered with the *N*-glycosylation of the ICAM-1 protein but did not markedly prevent its cell surface expression [25]. The structure–activity relationship of seven ursane-type triterpenoids showed that their inhibitory activities toward the cell surface expression and *N*-glycosylation of ICAM-1 were affected by the number of hydroxyl groups and/or the presence and position of a carboxyl group [27]. However, the structure–activity relationship of oleanane-type triterpenoids currently remains unclear. In the present study, the effects of 13 oleanane-type triterpenoids on cell viability and NF-κB activation and the protein expression, intracellular trafficking, and *N*-glycosylation of ICAM-1 in human lung adenocarcinoma A549 cells were evaluated.

The viability of A549 cells during a 7-h incubation was decreased by six triterpenoids at concentrations up to 100 µM. Two triterpenoids, i.e., celastrol (**12**) and pristimerin (**13**), possess one *α*,*β*-unsaturated carbonyl group. The *α*,*β*-unsaturated carbonyl groups common to celastrol (**12**) and pristimerin (**13**) appear to contribute to their cellular toxicity, possibly due to the chemical property of undergoing the Michael reaction in order to mediate a covalent modification to the cysteine residues of proteins [28,29]. Maslinic acid (**2**), hederagenin (**3**), echinocystic acid (**4**), and erythrodiol (**10**) also decreased the viability of A549 cells. These results are consistent with previous findings showing that the viability of A549 cells was decreased by maslinic acid (**2**) [30,31], hederagenin (**3**) [32,33,34], and echinocystic acid (**4**) [35]. Maslinic acid (**2**) and echinocystic acid (**3**) were also previously shown to induce apoptosis in A549 cells [30,31,35]. In contrast, erythrodiol (**10**) manifested negligible cytotoxicity against A549 cells at concentrations up to 100 µM [36]. Maslinic acid (**2**), hederagenin (**3**), echinocystic acid (**4**), and erythrodiol (**10**) possess two hydroxyl groups as a common structure. The presence of two hydrophilic hydroxyl groups in these triterpenoids increases their solubility and may be related to their cytotoxicity.

Glycyrrhetinic acid (**8**), celastrol (**12**), and pristimerin (**13**) possess one *α*,*β*-unsaturated carbonyl group. Celastrol (**12**) was shown to inhibit the activation of IκB kinase and TGF-β-activated kinase 1 upstream of the degradation of IκBα [37,38,39]. Pristimerin (**13**) was previously reported to prevent the degradation of IκBα [40,41]. Consistent with these findings, it was shown that celastrol (**12**) and pristimerin (**13**) inhibited the IL-1α-induced degradation of IκBα and its downstream nuclear translocation of the NF-κB subunit RelA in A549 cells. Unexpectedly, glycyrrhetinic acid (**8**) did not affect the IL-1α-induced expression of ICAM-1, suggesting that it does not inhibit the NF-κB signaling pathway. The *α*,*β*-unsaturated carbonyl groups in these three triterpenoids were present at different positions: the A ring in celastrol (**12**) and pristimerin (**13**) and the C ring in glycyrrhetinic acid (**8**). Therefore, the *α*,*β*-unsaturated carbonyl groups at the C ring appear to be very weakly reactive for cysteine residues via the Michael reaction. Synthetic unsaturated derivatives were shown to exhibit stronger anti-inflammatory activity than the parental glycyrrhetinic acid (**8**) by inhibiting the NF-κB and MAP kinase signaling pathways [42]. Therefore, oleanane-type triterpenoids have the potential to target NF-κB signaling proteins only when functional groups reactive to cysteine residues, such as an *α*,*β*-unsaturated carbonyl group, are present or are introduced at a suitable position.

Although oleanolic acid (**1**) did not markedly affect the cell surface expression of the ICAM-1 protein, it decreased its molecular size. This is consistent with our previous findings showing that oleanolic acid (**1**) interfered with the *N*-glycosylation of the ICAM-1 protein conjugated to shortened Endo H-resistant *N*-glycans and also that oleanolic acid directly inhibited the enzyme activity of yeast α-glucosidase [25]. The present study reported that moronic acid (**6**) and glycyrrhetinic acid (**8**) shortened the molecular size of the ICAM-1 protein under conditions that did not affect cell surface expression. In a similar manner to oleanolic acid (**1**) [25], shortened Endo-H-resistant *N*-glycans were attached to the ICAM-1 protein in A549 cells treated with moronic acid (**6**) and glycyrrhetinic acid (**8**). Moronic acid (**6**) and glycyrrhetinic acid (**8**) were recently shown to inhibit α-glucosidase activity [43,44,45]. Three glucose residues of the high-mannose-type *N*-glycans that attached to proteins were removed by ER α-glucosidases I and II, which were inhibited by castanospermine [46]. The similar effects of castanospermine and oleanolic acid (**1**) on the *N*-glycosylation and cell surface expression of the ICAM-1 protein indicate that oleanolic acid (**1**) targets ER α-glucosidases I and II [25]. Therefore, moronic acid (**6**) and glycyrrhetinic acid (**8**) also appear to interfere with the *N*-glycosylation of the ICAM-1 protein by inhibiting ER α-glucosidases I and II.

Among the 13 oleanane-type triterpenoids examined, it was shown that *α*-boswellic acid (**7**) selectively accumulated high-mannose-type *N*-glycans of the ICAM-1 protein, and this was accompanied by a reduction in the cell surface expression of the ICAM-1 protein. It was previously shown that maslinic acid (**2**) reduced the intracellular trafficking of the ICAM-1 protein, and this was accompanied by the accumulation of the high-mannose-type ICAM-1 protein [26]. The position of a carboxyl group and the number of hydroxyl groups differed between maslinic acid (**2**) and *α*-boswellic acid (**7**), indicating that they do not necessarily have a highly similar structure among the 13 oleanane-type triterpenoids tested. Although α-glucosidase activity was previously reported to be inhibited by maslinic acid (**2**) [26] and *α*-boswellic acid (**7**) [47], these inhibitory activities were not responsible for the appearance of the high-mannose-type (Endo-H-sensitive) *N*-glycans of the ICAM-1 protein. It was previously shown that ursane-type ursolic acid and asiatic acid induced the accumulation of the high-mannose-type *N*-glycans of ICAM-1 in the ER by inhibiting protein trafficking to the Golgi apparatus [11,25]. However, the molecular mechanisms by which ursolic acid and asiatic acid interfere with protein trafficking from the ER to the Golgi apparatus remain unclear. It was demonstrated that ursane-type *β*-boswellic acid, a structural isomer of α-boswellic acid (**7**), induced the accumulation of a shorter ICAM-1 protein, which conjugated to both Endo-H-sensitive and Endo-H-resistant *N*-glycans in a manner that differed from that of other ursane-type triterpenoids [27]. Therefore, further experiments are needed to identify the target proteins of oleanane- and ursane-type triterpenoids, which may be involved in protein trafficking in the ER to the Golgi apparatus.

**Figure 10:**
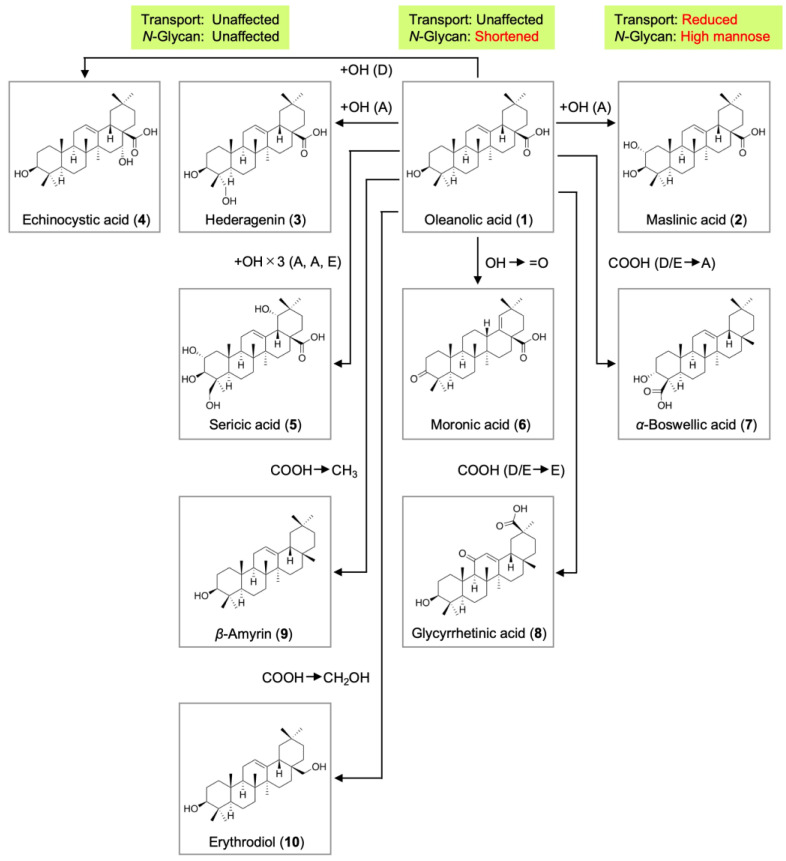
Structure–activity relationship of oleanane-type pentacyclic triterpenoids. Hederagenin (**3**), echinocystic acid (**4**), sericic acid (**5**), *β*-amyrin (**9**), and erythrodiol (**10**) did not affect the transport of the ICAM-1 protein to the cell surface or *N*-glycan modifications to the ICAM-1 protein under conditions where cell viability was not diminished. Oleanolic acid (**1**), moronic acid (**6**), and glycyrrhetinic acid (**8**) did not exert any effects on the transport of the ICAM-1 protein to the cell surface but affected the *N*-glycosylation of the ICAM-1 protein by shortening Endo-H-resistant *N*-glycans. Maslinic acid (**2**) and *α*-boswellic acid (**7**) reduced the transport of the ICAM-1 protein to the cell surface and interfered with the *N*-glycosylation of the ICAM-1 protein, resulting in the accumulation of Endo-H-sensitive high-mannose-type *N*-glycans. Differences in hydroxyl and carboxyl groups from oleanolic acid are indicated by arrows. The positions of hydroxyl groups attached to the A to E rings are indicated in parentheses.

Based on the present results and previous findings, the structure–activity relationship of oleanane-type triterpenoids is summarized in Figure 10. Hederagenin (**3**), echinocystic acid (**4**), sericic acid (**5**), *β*-amyrin (**9**), and erythrodiol (**10**) did not markedly affect the transport or *N*-glycosylation of the ICAM-1 protein at concentrations that did not decrease cell viability. Oleanolic acid (**1**), moronic acid (**6**), and glycyrrhetinic acid (**8**) shortened the *N*-glycans of ICAM-1, possibly by inhibiting ER α-glucosidases I and II. Maslinic acid (**2**) and α-boswellic acid (**7**) induced the accumulation of high-mannose-type *N*-glycans of ICAM-1 and reduced the transport of the ICAM-1 protein to the cell surface. Overall, the inhibitory activities of oleanane-type triterpenoids against protein transport and *N*-glycosylation were affected by the presence and position of the carboxyl group and the number and position of hydroxyl groups. The carboxyl group is required for the inhibitory activities of oleanane-type triterpenoids. Maslinic acid (**2**), hederagenin (**3**), and echinocystic acid (**4**) are three structural isomers (H_30_H_48_O_4_) that possess an additional hydroxyl group at different positions from that in oleanolic acid (**1**). Based on the present results showing that these isomers manifested different inhibitory profiles, it is proposed that the position of hydroxyl groups is important for assessing the inhibitory activities of oleanane-type triterpenoids.

## 4. Materials and Methods

### 4.1. Cell Culture

Human lung adenocarcinoma A549 cells (JCRB0076; National Institutes of Biomedical Innovation, Health and Nutrition JCRB Cell Bank, Osaka, Japan) were maintained in RPMI 1640 medium (Thermo Fisher Scientific, Gland Island, NY, USA) supplemented with fetal calf serum (Sigma-Aldrich, St. Louis, MO, USA) treated at 56 °C for 30 min and a penicillin–streptomycin antibiotic mixture (Nacalai Tesque, Kyoto, Japan). A549 cells were subcultured every 2 to 3 days and dispersed onto plates or dishes one day before experiments. The medium was replaced with new medium prior to the treatment with triterpenoids.

### 4.2. Reagents

Thirteen oleanane-type pentacyclic triterpenoids, oleanolic acid (**1**), maslinic acid (**2**), hederagenin (**3**), echinocystic acid (**4**), sericic acid (**5**), moronic acid (**6**), α-boswellic acid (**7**), glycyrrhetinic acid (**8**), β-amyrin (**9**), erythrodiol (**10**), gymnemagenin (**11**), celastrol (**12**), and pristimerin (**13**), were obtained from commercial suppliers (Table S1). IL-1α was kindly provided by Dainippon Pharmaceutical (Osaka, Japan).

### 4.3. Antibodies

Primary mouse antibodies to β-actin (AC-15; Sigma-Aldrich, St. Louis, MO, USA), γ1-actin (2F3; Fujifilm-Wako Pure Chemical Corporation, Osaka, Japan), GAPDH (6C5; Santa Cruz Biotechnology, Dallas, TX, USA), ICAM-1 (28; BD Bioscience, San Diego, CA, USA), IκBα (25/IkBa/MAD-3; BD Biosciences, San Jose, CA, USA), lamin A/C (E-1; Santa Cruz Biotechnology, Dallas, TX, USA), and RelA (F-6; Santa Cruz Biotechnology, Dallas, TX, USA) were used for Western blotting. A mouse anti-ICAM-1 antibody (15.2; Leinco Technologies, Inc., St. Louis, MO, USA) was used for cell ELISA. Peroxidase-conjugated goat anti-mouse IgG (H + L) (Jackson ImmunoResearch Laboratories, West Grove, PA, USA) was used as a secondary antibody for Western blotting and cell ELISA.

### 4.4. Evaluation of Cell Viability

The 3-(4,5-(dimethyl-2-thiazolyl)-2,5-diphenyl-2H-tetrazolium bromide (MTT) assay was used to evaluate cell viability. MTT is converted to blue MTT formazan by cellular reducing activity [48]. A549 cells (2 × 10^4^ cells/well) were dispersed in 96-well plates and then precultured overnight. A549 cells were treated with triterpenoids for 7 h and incubated with MTT (0.45 mg/mL) for the last 2 h of the incubation. MTT formazan was solubilized in 4.8% sodium dodecyl sulfate (SDS) overnight. The iMark^TM^ microplate reader (Bio-Rad Laboratories, Hercules, CA, USA) was used to measure absorbance at 570 nm. Cell viability (%) was calculated using the following formula: (test − background)/(control − background) × 100.

### 4.5. Preparation of Cell Lysates

A549 cells were dispersed in 12-well plates (2 × 10^5^ cells/well), 6-well plates (5 × 10^5^ cells/well), or 35 mm dishes (5 × 10^5^ cells/dish) and then precultured overnight. A549 cells were treated with triterpenoids for 1 h and then stimulated with IL-1α for 15 min, 1 h, or 6 h. A549 cells were washed once with ice-cold phosphate-buffered saline (PBS) and treated with Triton X-100 lysis buffer consisting of 1% Triton X-100, 50 mM Tris-HCl (pH 7.5), 2 mM dithiothreitol, 2 mM sodium vanadate, and a cOmplete^TM^ protease inhibitor cocktail (Sigma-Aldrich, St. Louis, MO, USA) on ice for 15 min. After centrifugation (15,300× *g*, 4 °C, 5 min), supernatants were recovered as cell lysates or cytoplasmic fractions. Precipitates were washed with Triton X-100 lysis buffer, treated with sonication, and collected as nuclear fractions. The protein content of cell lysates was assessed using Protein Assay CBB Solution (5×) (Nacalai Tesque, Kyoto, Japan).

### 4.6. Evaluation of Protein Expression

Cell lysates adjusted to an equal protein content were mixed with 3× sample buffer consisting of 187.5 mM Tris, 6% SDS, 30% glycerol, 0.009% bromophenol blue, and 864 mM 2-mercapthoethanol, heated at 100 °C for 5 min and then separated by SDS-polyacrylamide gel electrophoresis (PAGE). Proteins were transferred onto a ClearTrans^®^ nitrocellulose membrane, 0.2 µm (Fujifilm Wako Pure Chemical Corporation, Osaka, Japan), by Mini Trans-Blot^®^ Cell (Bio-Rad Laboratories, Hercules, CA, USA). Membranes were blocked with 0.5% Tween 20 in PBS (PBS-T) containing 5% skim milk overnight at 4 °C. Primary and secondary antibodies were diluted with 5% skim milk in PBS-T and then reacted with the membranes at room temperature for 1 h. After the antibody reaction, the membranes were washed with PBS-T. Amersham^TM^ ECL^TM^ Western blotting detection reagents (GE Healthcare Japan, Tokyo, Japan) and ImmunoStar^®^ Zeta (Fujifilm Wako Pure Chemical Corporation, Osaka, Japan) were used for the peroxidase-based chemiluminescence reaction. Amersham Imager 680 (GE Healthcare Japan, Tokyo, Japan) was used to acquire protein bands. Images were further analyzed by ImageQuant TL software 7.0.1.0 (GE Healthcare Japan, Tokyo, Japan). After the first round of Western blotting, the membranes were treated with stripping solution (Fujifilm Wako Pure Chemical Corporation) and blocked with 5% skim milk in PBS-T. Membranes were further treated with an anti-β-actin antibody, anti-γ1-actin antibody, anti-lamin A/C antibody, or anti-GAPDH antibody as loading controls.

### 4.7. Evaluation of Cell Surface ICAM-1 Protein Expression

A549 cells (2 × 10^4^ cells/well) were dispersed in 96-well plates and then precultured overnight. A549 cells were treated with triterpenoids for 1 h and then stimulated with IL-1α for 6 h. A549 cells were washed once with PBS and then fixed with 1% paraformaldehyde in PBS for 15 min. Fixed cells were washed three times with PBS and blocked with 1% bovine serum albumin (BSA) in PBS at 4 °C overnight. The primary mouse anti-ICAM-1 antibody (15.2) and secondary peroxidase-conjugated goat anti-mouse IgG (H + L) were diluted with 1% BSA in PBS and reacted with cells at room temperature for 1 h. After the antibody reaction, cells were washed three times with 0.02% Tween 20 in PBS. The substrate solution (0.2 M citrate buffer (pH 5.3), 0.1% *o*-phenylene diamine, and 0.02% hydrogen peroxide) was used for the peroxidase reaction at room temperature. The iMark^TM^ microplate reader was used to measure absorbance at 450 nm. ICAM-1 expression (%) was calculated by the following formula: (test − background)/(IL-α treatment (positive control) − background) × 100.

### 4.8. Evaluation of N-Linked Glycans by Glycosidases

Cell lysates were prepared as described in Section 4.5. According to the manufacturer’s protocols, cell lysates were heated at 100 °C for 10 min, and the resultant cell lysates were treated with PNGase F (New England BioLabs, Ipswich, MA, USA) and Endo H (New England BioLabs, Ipswich, MA, USA) at 37 °C for 1 h. Cell lysates were then treated with 3× sample buffer and analyzed by SDS-PAGE and Western blotting as described in Section 4.6.

### 4.9. Data Analysis

All experiments were independently repeated at least three times. In the quantitative analysis, data are presented as the mean ± S.E. of at least three independent experiments. A one-way ANOVA and Tukey’s post hoc test were used to evaluate the significance of differences. *p* values less than 0.05 were regarded as significant.

## 5. Conclusions

Oleanane-type triterpenoids are known to inhibit the various cellular proteins responsible for anti-inflammatory and anti-cancer activities. ICAM-1 plays an essential role in inflammation and cancer metastasis. The present study investigated the structure–activity relationship of 13 oleanane-type triterpenoids on the NF-κB signaling pathway and on the expression, cell surface transport, and *N*-glycosylation of the ICAM-1 protein. Two triterpenoids possessing an *α*,*β*-unsaturated carbonyl group, celastrol (**12**) and pristimerin (**13**), inhibited the NF-κB signaling pathway. Three triterpenoids, oleanolic acid (**1**), moronic acid (**6**), and glycyrrhetinic acid (**8**), interfered with the *N*-glycosylation of the ICAM-1 protein without affecting its cell surface transport. Two triterpenoids, maslinic acid (**2**) and *α*-boswellic acid (**7**), interfered with the cell surface transport of the ICAM-1 protein and promoted the accumulation of high-mannose-type *N*-glycans. These processes were inhibited by oleanane-type triterpenoids in a manner that depended on their overall structure and functional groups, including carboxyl, hydroxyl, and *α*,*β*-unsaturated carbonyl groups. Further studies are needed to elucidate the molecular mechanisms and target proteins of oleanane-type pentacyclic triterpenoids.

## Data Availability

The data presented in this study are available upon reasonable request from the corresponding author.

## Supplementary Materials

The following supporting information may be downloaded at https://www.mdpi.com/article/10.3390/ijms25116026/s1.
