# Supplementary material for: Structure–Activity Relationship of Oleanane-Type Pentacyclic Triterpenoids on Nuclear Factor κB Activation and Intracellular Trafficking and N-Linked Glycosylation of Intercellular Adhesion Molecule-1
Source: Int J Mol Sci. 2024 May 30;25(11):6026. doi: 10.3390/ijms25116026 (PMC11173061; doi:10.3390/ijms25116026)
Supplement: Supplementary file 1 [file ijms-25-06026-s001.zip › ijms-2991403-supplementary.pdf]

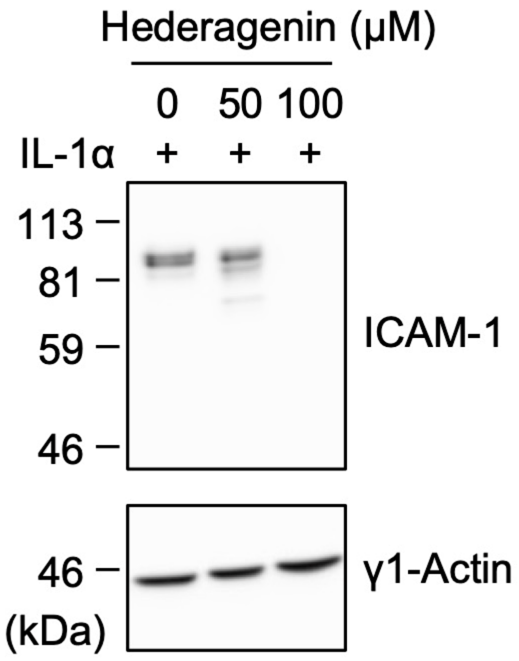

Figure S1. Effects of hederagenin on the IL-1α-induced ICAM-1 protein expression. A549 cells were treated with or without hederagenin (**3**) for 1 h, and were then stimulated with (+) IL-1α (0.25 ng/mL) for 6 h in the presence or absence of hederagenin (**3**) at the indicated final concentrations. Blots are representative of three independent experiments.

Table S1. Oleanane-type pentacyclic triterpenoids used in this study

No.	Compound	Molecular formula	Supplier	Product number	Batch (Lot) number	Purity (%)
**1**	Oleanolic acid	$C_{30}H_{48}O_3$	Sigma-Aldrich	05504-100MG	SLBD3309V	≥ 97
**2**	Maslinic acid	$C_{30}H_{48}O_4$	Cayman Chemical	10009645	0480403-12	≥ 98
**3**	Hederagenin	$C_{30}H_{48}O_4$	Tokyo Chemical Industry	H1645	QZFAM-LM	≥ 98
**4**	Echinocystic acid	$C_{30}H_{48}O_4$	Cayman Chemical	27476	0555516-1	≥ 95
**5**	Sericic acid	$C_{30}H_{48}O_6$	Santa Cruz Biotechnology	SC-396534	J2919	≥ 98
**6**	Moronic acid	$C_{30}H_{46}O_3$	Tokyo Chemical Industry	M2610	GVFCB-KF	≥ 96
**7**	*α*-Boswellic acid	$C_{30}H_{48}O_3$	Cayman Chemical	11692	0545283-1<br>0585229-4	≥ 98<br>≥ 98
**8**	Glycyrrhetinic acid	$C_{30}H_{46}O_4$	Tokyo Chemical Industry	G0149	JJVRC-NM	≥ 97
**9**	*β*-Amyrin	$C_{30}H_{50}O$	Cayman Chemical	20949	0536677-6	≥ 95
**10**	Erythrodiol	$C_{30}H_{50}O_2$	Sigma-Aldrich	09258-10MG-F	BCBH3524V	≥ 97
**11**	Gymnemagenin	$C_{30}H_{50}O_6$	Cayman Chemical	11713	0510955-2	≥ 98
**12**	Celastrol	$C_{29}H_{38}O_4$	Cayman Chemical	70950	0507248-19	≥ 98
**13**	Pristimerin	$C_{30}H_{40}O_4$	Cayman Chemical	13621	0476159-13	≥ 98
